# Supplementary material for: Biocontrol Potential of Endophytic Bacillus velezensis LSR7 Against Rubber Red Root Rot Disease
Source: J Fungi (Basel). 2024 Dec 9;10(12):849. doi: 10.3390/jof10120849 (PMC11678668; doi:10.3390/jof10120849)
Supplement: Supplementary file 1 [file jof-10-00849-s001.zip › jof-3298526-supplementary.pdf]

Table S1. PCR reaction system of endophytic bacteria LSR7

Gene sequence	Reaction System	Amount of reaction
-	2×Taq PCR Master Mix (Vazyme)	25 μL
16S rDNA	27F (5′ - AGAGTTTGATCCTGGCTCAG -3′)	1 μL
	1492R (5′ - GGTTACCTTGTTACGACTT -3′)	1 μL
gyrB gene	UP1 (5′-GAAGTCATCATGACCGTTCTGCAYGCNGGNGGNAARTTYGA-3′)	1 μL
	UP2r (5′-AGCAGGGTACGGATGTGCGAGCCRTCNACRTCNGCRTCNGTCAT-3′)	1 μL
-	DNA template	2 μL
-	dd H_2O	21μL

Table S2. PCR reaction procedure of endophytic bacteria LSR7

Reaction conditions	Reaction time	Number of cycles
95°C (Predenaturation)	3 min	
95°C	1 min	
58°C	1 min	35
72°C	1 min 30 s	
72℃(Terminal extension)	10 min	
4°C	store	

Table S3. qPCR reaction system of endophytic bacteria LSR7

Reaction System	Amount of reaction
2× Q3 SYBR qPCR Master Mix (Cellagen Technology, San Diego, CA, USA)	10.0 μL
ACT (forward: 5′-CATCGAGCACGGTATTGTCA-3′)	0.5 μL
ACT (reverse: 5′-TCTCGAACATGATTTGGGTC-3′)	0.5 μL
18S rRNA (forward: 5′-ACGAAGGTTAGGGGATCGAAA-3′)	0.5 μL
18S rRNA (reverse: 5′-CGAGCGACACATAAGATTGAGG-3′)	0.5 μL
DNA template	2 μL
dd H_2O	7 μL

Table S4. qPCR reaction system of endophytic bacteria LSR7

Reaction conditions	Reaction time	Number of cycles
95°C (Predenaturation)	3 min	
95°C	30 s	
55°C	30 s	35
72°C	1 min s	
72℃(Terminal extension)	10 min	
4°C	store	

Table S5. Genome feature of strain LSR7

**Type**	**Number**	**Total_len**	**Average_len**	**Percentage of genome (%)**
Gene	3,908	3,517,808	900	90.01
CDS	3,711	34,575,554	932	88.47
tRNA	86	6,665	78	0.17
23S rRNA	9	26,334	2,926	0.67
16S rRNA	9	13,923	1,547	0.36
5S rRNA	9	999	111	0.03
tm RNA	1	360	360	0.01

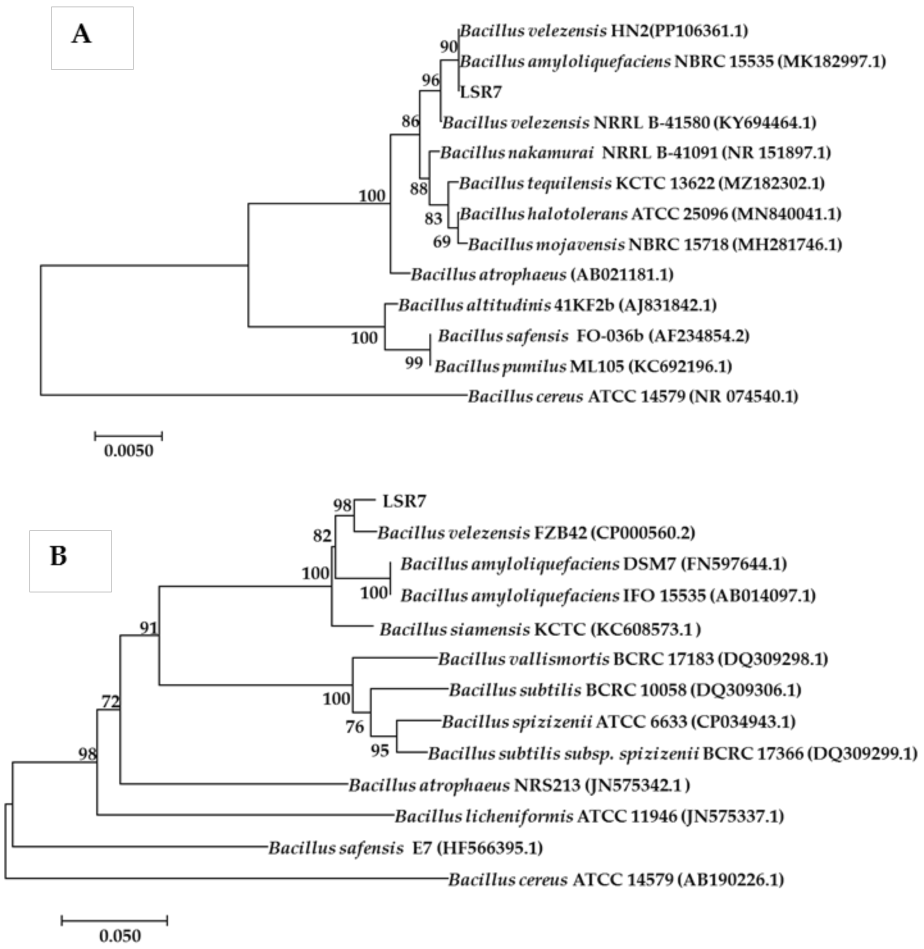

Figure S1. Phylogenetic trees based on the 16S rDNA sequence (A) and *gyrB* sequence (B) of LSR7 and their homologous sequences. Phylogenetic trees were constructed by the Neighbor-joining method of MEGA 7.0 with bootstrap values based on 1000 replications. *B. cereus* ATCC 14579 was chosen as the out-group. Gene bank accession numbers of bacterial strains are shown in parentheses. The scale bar represents the number of substitutions per base position.

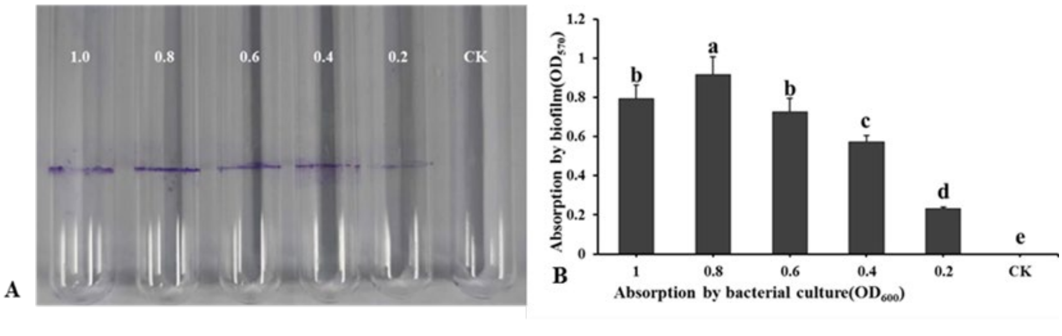

Figure S2. Biofilm formation. **(A)** Biofilms developing on glass tubes were stained with 0.1% crystal violet. (For interpretation of the references to colour in this figure legend, the reader is referred to the web version of this article). **(B)** The mean amount of biofilm produced by different concentrations culture (OD_{600} = 0.2, 0.4, 0.6, 0.8, 1.0). Error bars represent the standard error of the average value, and the letter denotes significant differences at $p < 0.05$.

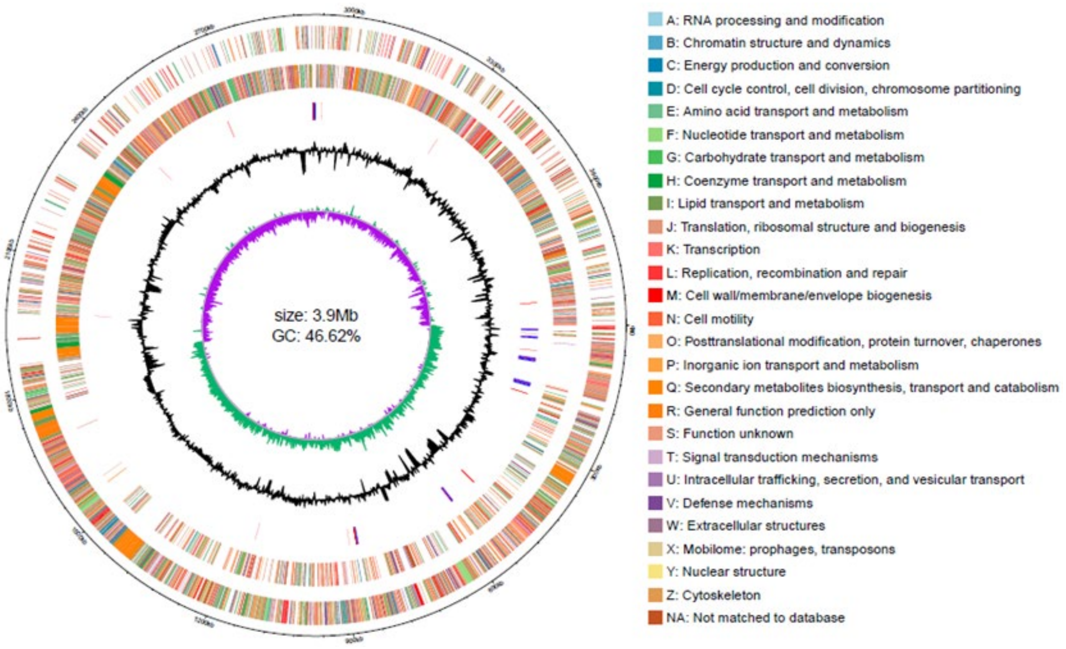

Figure S3. Circular map of strain LSR7 genome

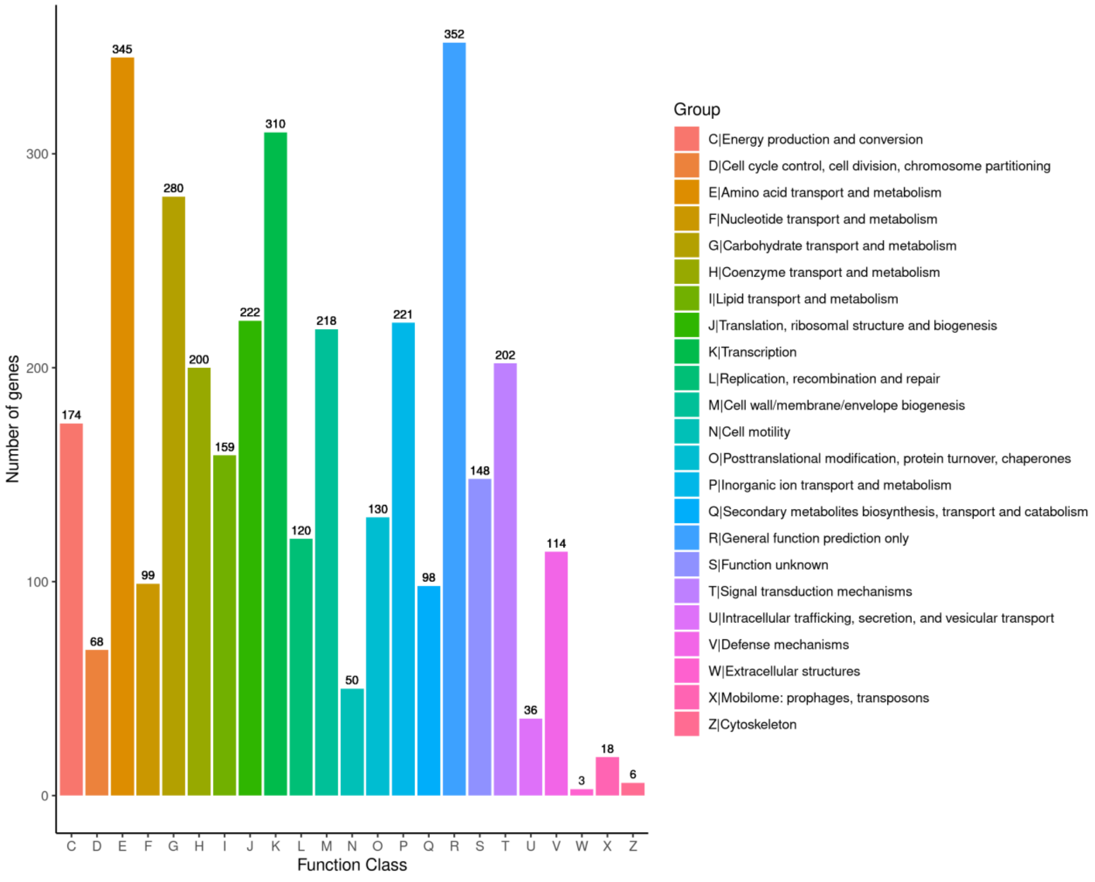

Figure S4. COG annotation of the strain LSR7 genome

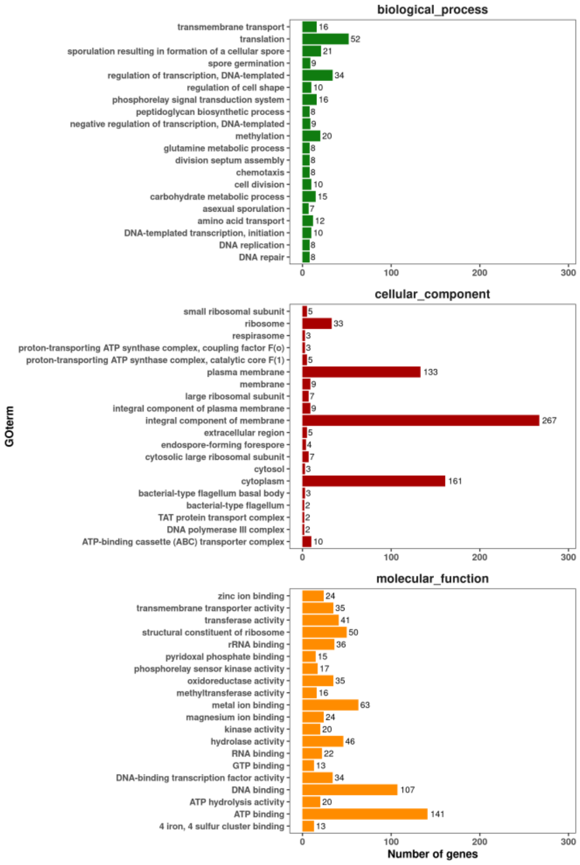

Figure S5. GO annotation of the strain LSR7 genome

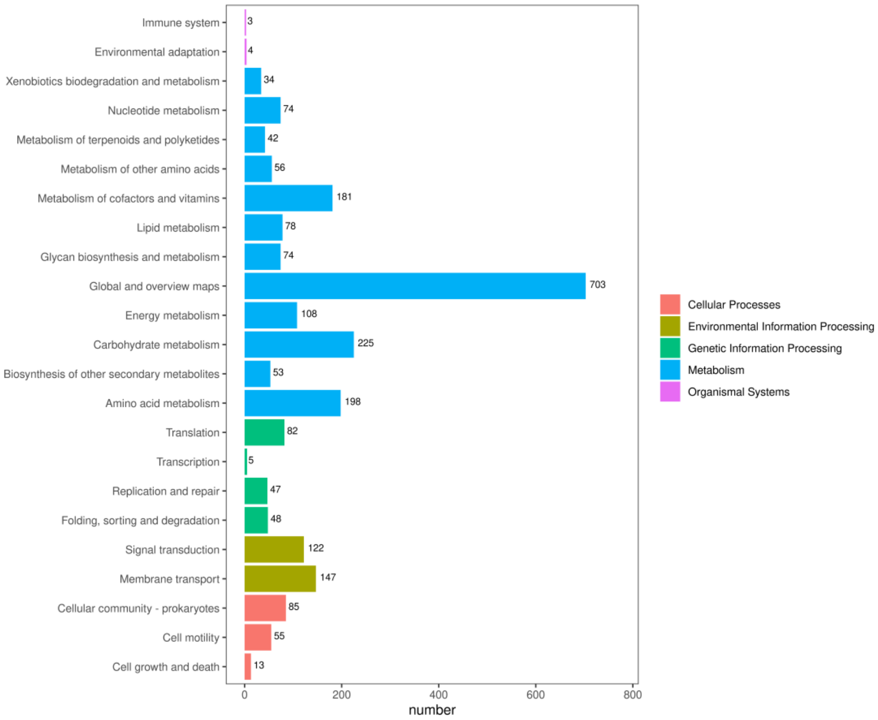

Figure S6. KEGG passageway annotation of the strain LSR7 genome

Region	Type	From	To	Most similar known cluster		Similarity
Region 1.1	NRPS	304,769	370,176	surfactin	NRP:Lipopeptide	82%
Region 1.2	thiopeptide , LAP	571,106	600,219	kijanimicin	Polyketide	4%
Region 1.3	RRE-containing , LAP	686,677	709,854	plantazolicin	RiPP:LAP	91%
Region 1.4	PKS-like	921,711	962,955	butirosin A/butirosin B	Saccharide	7%
Region 1.5	terpene	1,044,996	1,065,736			
Region 1.6	transAT-PKS	1,370,516	1,458,725	macrolactin H	Polyketide	100%
Region 1.7	transAT-PKS , T3PKS , NRPS	1,680,080	1,790,203	bacillaene	Polyketide+NRP	100%
Region 1.8	NRPS , transAT-PKS , betalactone	1,852,134	1,989,968	fengycin	NRP	100%
Region 1.9	terpene	2,012,534	2,034,417			
Region 1.10	T3PKS	2,105,183	2,146,283			
Region 1.11	transAT-PKS	2,261,793	2,367,962	difficidin	Polyketide	100%
Region 1.12	NRPS	2,855,620	2,899,573	bacillothiazol A/bacillothiazol B/bacillothiazol C/bacillothiazol D/bacillothiazol E/bacillothiazol F/bacillothiazol G/bacillothiazol H/bacillothiazol I/bacillothiazol J/bacillothiazol K/bacillothiazol L/bacillothiazol M/bacillothiazol N	NRP	100%
Region 1.13	NRP-metallophore , NRPS , RiPP-like	3,000,164	3,051,957	bacillibactin	NRP	100%
Region 1.14	other	3,563,166	3,604,584	bacilysin	Other	100%

Figure S7. Prediction information of BCGs in strain LSR7 genome by antiSMASH online tool (https://antismash.secondarymetabolites.org/).

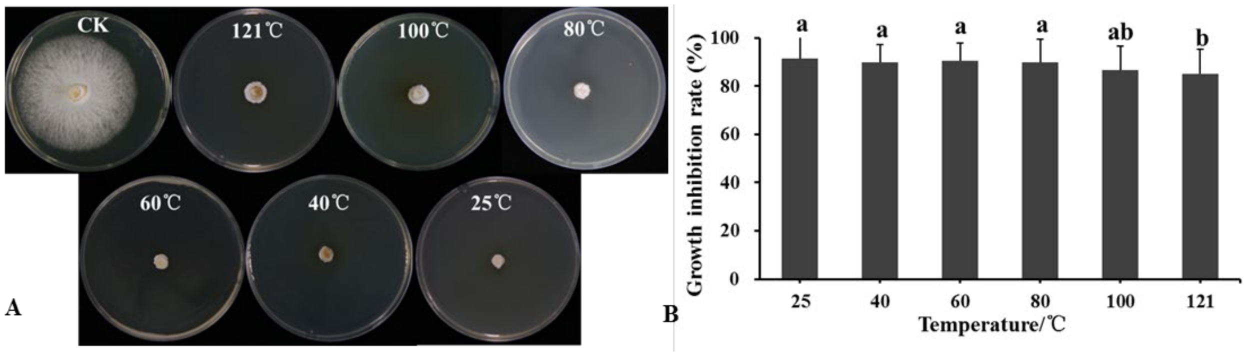

Figure S8. (A, B) Effect of temperature on antifungal activity of LSR7. Bars indicate the standard error of the mean. Columns marked with the same letter are not significantly different according to Duncan's Multiple Range Test at $p<0.05$.
